# IPDT Model-Based Ziegler–Nichols Tuning Generalized to Controllers with Higher-Order Derivatives

**DOI:** 10.3390/s23083787

**Published:** 2023-04-07

**Authors:** Pavol Bistak, Mikulas Huba, Damir Vrancic, Stefan Chamraz

**Affiliations:** 1 Institute of Automotive Mechatronics, Faculty of Electrical Engineering and Information Technology, Slovak University of Technology in Bratislava, SK-812 19 Bratislava, Slovakia; 2 Department of Systems and Control, J. Stefan Institute, SI-1000 Ljubljana, Slovenia

**Keywords:** filtration, automatic reset, hyper reset, stability, robustness, multiple real dominant pole method, derivative action, constrained control

## Abstract

The paper extends the earlier work entitled “Making the PI and PID Controller Tuning Inspired by Ziegler and Nichols Precise and Reliable”, to higher-order controllers and a broader range of experiments. The original series PI and PID controllers, based on automatic reset calculated by filtered controller outputs, are now augmented by higher-order output derivatives. This increases the number of degrees of freedom that can be used to modify the resulting dynamics, accelerates transient responses, and increases robustness to unmodeled dynamics and uncertainties. The fourth order noise attenuation filter used in the original work allows for the addition of an acceleration feedback signal, thus resulting in a series PIDA controller or even a jerk feedback that leads to a PIDAJ series controller. Such a design can further use the original process and filter approximation of the step responses through the integral-plus-dead-time (IPDT) model, while allowing experimentation with disturbance and setpoint step responses of the series PI, PID, PIDA and PIDAJ controllers, and thus, evaluating the role of output derivatives and noise attenuation from a broader perspective. All controllers considered are tuned using the Multiple Real Dominant Pole (MRDP) method, which is complemented by a factorization of the controller transfer functions to achieve the smallest possible time constant for automatic reset. The smallest time constant is chosen to improve the constrained transient response of the considered controller types. The obtained excellent performance and robustness allow the proposed controllers to be applied to a wider range of systems with dominant first-order dynamics. The proposed design is illustrated on a real-time speed control of a stable direct-current (DC) motor, which is approximated (together with a noise attenuation filter) by an IPDT model. The transient responses obtained are nearly time-optimal, with control signal limitations active for most setpoint step responses. Four controllers with different degrees of derivative with generalized automatic reset were used for comparison. It was found that controllers with higher-order derivatives may significantly improve the disturbance performance and virtually eliminate overshoots in the setpoint step responses in constrained velocity control.

## 1. Introduction

The rapid development of the technological base of automatic control in terms of digital and hybrid programmable devices and embedded systems, actuators, sensors, digital communications and signal processing also requires a thorough reassessment of the historical development of the most widely used control structure, the proportional–integral–derivative (PID) controller. Such a review should include all related steps, starting with the modeling and identification of the controlled process and relating to all important aspects of the design, including process uncertainties, measurement noise and system nonlinearities. At the same time, an important aspect related to the term used should also be addressed, namely explaining the differences between controllers that compensate for disturbances and uncertainties by an explicit integrator or by an automatic reset.

The first known method of controller tuning, by Ziegler and Nichols [1], appeared shortly after the invention of hyper-reset (pre-act) controllers (controllers with derivative term) [2]. In the original version, the mentioned tuning was mainly aimed at the fastest possible elimination of the control error due to the occurrence of disturbances. It was less suitable for setpoint tracking, since it usually led to overshooting of the process output (overregulation) [3].

The improvements presented in [4] were achieved by (a) a modified approximation of the open-loop step responses (using alternatively both the integrating and the stable first-order process models with delay), (b) designing the controller parameters according to the multiple real dominant poles method with subsequent factorization of the controller numerator improving constrained control responses (using the series realization of the PID controller), (c) an appropriate choice of the time constant for the automatic reset (the smallest numerator time constant) [5] and (d) replacing the requirements of quarter-amplitude damping by shape-related performance measures based on monotonicity. This choice of the time constant of the equivalent “integration” and the series realization of the PID controller with 2 degrees of freedom, which is unusual in general, proved to be efficient for constrained processes. It allowed the elimination of overshoots in the constrained control and the achievement of transient responses close to the ideal ones (optimal switching control). This means that the responses are fast enough and without overshoot of the output during the setpoint change. It is worth noting that the more sophisticated stable first-order time-delayed (FOTD) models did not significantly improve the control performance in our experiments.

In [4], the focus was on the optimization of the setpoint tracking, while the disturbance performance is also very important, especially for the closed-loop robustness. In [6], it was shown that in terms of robust stability, and when using controllers with higher-order low-pass filters, the best approximation of the IPDT model is the one with the highest process gain.

Another significant advantage of the series controller realized by the feedback of the limited controller output is that such feedback can also be interpreted as a disturbance observer based on the reconstruction of input disturbances in steady states [7,8]. Of course, the information about the compensated disturbance is also available from the integral term of the parallel PI (D) controllers. However, its direct use is complicated by limiting the output of the controller.

Research in the field of PID controllers is strong and expressed through special IFAC (International Federation of Automatic Control) conferences and events. The review of papers from the past (2018) Conference on Advances in Proportional–Integral–Derivative Control in Ghent showed increased interest from the control community in fractional-order PID controllers (FO). FO PID control provides a higher number of degrees of freedom in the design phase [9,10]. However, it ultimately leads to the final implementation of higher-order controllers (HO). It is, therefore, understandable that there is increasing research interest in PID controllers with HO derivatives, e.g., called proportional–integral–derivative–accelerative (PIDA) controllers [11,12,13,14,15,16,17,18,19,20], or also PIDD2, PIDD${}^{2}$ [20,21,22,23,24], or PIDC (PIDC1 and PIDC2) [19]. Therefore, this paper will focus on combining the previously mentioned aspects of modified controller design inspired by the Ziegler and Nichols method with higher-order controller derivatives. The first PIDA controllers were designed for optimal control of systems with three or more elementary energy or mass storage devices, which is reflected in the third- or higher-order process transfer function [11,12,13,14,21,25,26]. Relevant applications include load frequency control of power systems [19,22,25,26], automatic voltage regulator (AVR) of a synchronous generator [14,20,21], flight control of a quadcopter [18], a dual rotor aerodynamic system [23], drones [18], DC–DC boost converters [24], three-tank control [26], etc. With respect to the design of state–space controllers, the order of the process determines the required number of control vector coefficients, which may increase if signals are needed for disturbance reconstruction. Note that for time-delayed systems, the order of the system under consideration also depends on the degree of the dead-time approximation used in Taylor series expansion [27,28,29].

The number of HO PID controller realizations grows with the number of possible realizations of higher derivative orders and optimization of the number of controller parameters [28]. By default, separate minimum order filters are used for each derivative term when designing PID controllers as well as controllers with higher-order derivatives. With such an approach, as the derivative degree *m* increases, the number of unknown parameters increases and the delays of the individual derivative terms change. This complicates the design of a suitable noise reduction scheme. Therefore, from the point of view of the analytical design of the optimal controller, it is easier to consider a joint binomial series filter. The high-frequency controller noise can be attenuated most effectively by strict proper controller transfer functions. A simple experimental analysis in [30], confirmed by several subsequent publications on the subject, showed that the filter order n≥m+2 can be recommended as the default option. The filter parameters were considered in the controller tuning as part of the loop delay using simple delay equivalences inspired by Skogestad’s half rule [31]. A further simplification, based on the work of Ziegler and Nichols [1], can be achieved by approximating the (stable, but possibly unstable) process with time-delay integral models. The comparison in [32] shows that the influence of the neglected first-order dominant process dynamics decreases as the derivative order of the controller increases. Another alternative was to include the binomial filter already in the measurement and in the approximation of the open-loop step response [4]. Due to the large number of unknown controller parameters, the design using traditional engineering methods (such as root locus in [11,12]) was imperfect even when the filter parameters were neglected. As a result, recent research in HO PID control has focused more on demonstrating various intelligent optimization algorithms. This has certainly given the impression of modernity, but generalization of the obtained results is negligible. Although the aforementioned optimization approaches can evolve, it is always useful to reduce the complexity of the solved problems by searching for more generally applicable analytical laws and procedures.

In the article [27], it was shown that using the Multiple Real Dominant Pole (MRDP) method, it is possible to reliably design parallel HO-PID controllers with derivatives up to order m=5, taking into account the binomial implementation and noise attenuation filters of order n≥m.

Other problems are related to the limitation of the control signal, which usually cannot be avoided when accelerating transient responses. To avoid undesirable integration of the controller output in the presence of limited control signals, parallel HO-PID controllers [27] could be implemented using the conditional technique [33]. Due to the 2-degree-of-freedom control, the input and output signals of the process have some overshoots, which are not allowed in numerous applications. For this reason, ref. [4] has raised the problem of constrained series PI and PID controllers following the method of Ziegler and Nichols, and ref. [28] has extended the problem to the design of series PIDA controllers that eliminate overshoots at the process input and output, which occur in saturation control, by appropriate controller factorization and by the performance portrait method. This paper focuses on the further generalization of the controllers with disturbance compensation by automatic resetting and the output derivatives up to the third order. An experimental comparison of the setpoint and disturbance behavior of controllers with increasing derivative ratio is illustrated using an electromechanical system from [4]. It is shown how the idea of modifying the transfer function of series PID controller by factorization can also be applied to the design of a constrained HO.

In this context, the rest of the paper is organized as follows. Section 2 summarizes the main previous results of the experimental design of series PI and PID controllers for the IPDT model of a speed servo system from [4], and proposes its extension to controllers with second and third output derivatives. Section 3 summarizes the tuning of the series PI and PID controllers by the multiple real dominant pole (MRDP) method and its extension by a prefilter from [4], and extends the problem to the series PIDA and PIDAJ controllers with a simple disturbance-observer-based automatic reset for disturbance reconstruction and a compensation by a positive feedback (automatic reset) from the controller output. This section also contains a brief analysis of the problems caused by the saturation of the control signal, which led to the application of the circle criterion of absolute stability. The main results of the experimental evaluation of series PI and PID controllers based on the speed servo system from [4], extended to include disturbance responses and the use of the PIDA and PIDAJ controllers, are presented in Section 4 and discussed in Section 5. The conclusions include a summary of the work and future developments.

## 2. Controller Structure and Process Approximation

The main contribution of the original article [4] can be identified as the overall design of the constrained series PID controller, which included:

1. The justification of the windupless controller structure (denoted originally as hyper-reset, or pre-act [2]) using a positive feedback from the constrained controller output for resetting the PD controller output;
2. The approximation of the fastest component of the measured open-loop step response, dominating its steepest segment by the “ultralocal” IPDT, the more complex “local” first-order time-delayed (FOTD) models did not bring significant differences in the final performance achieved;
3. The calculation of the “optimal” controller parameters by the multiple real dominant pole (MRDP) method based on the IPDT and FOTD models, including the noise attenuation and implementation filter used;
4. Factorization of the controller transfer function corresponding to the feedback structure obtained in the zone of proportional control, carried out with the aim of choosing the smallest possible time constant of the hyper-reset feedback;
5. A demonstration of the properties of the proposed design by control of the electro-mechanical speed servo system using evaluation by means of appropriately chosen time and shape-related performance measures.

**Remark** **1** (Windupless controller). *Because controllers with automatic reset and hyper reset do not contain an explicit integrator, there is no point in talking about anti-windup structures. The negative consequences of limiting the control signal to the form of transient responses, resulting into overshooting at the process input and output, can be simply remedied by suitable controller settings. This will be achieved by choosing the reset time constant as the shortest time constant of the “optimal” controller numerator calculated by the multiple real dominant pole method.*

This contribution aims to show that the procedure applied in [4] can be relatively easily extended to the design of the entire family of constrained windupless controllers with higher-order derivatives and automatic reset. All controllers with derivative action offer excellent properties, which surpass the dynamics achievable by well-known anti-windup controllers based on the parallel HO PID controllers discussed in [27]. In order to save the reader as much as possible from the study of new facts, the experiment from [4] will again be used to demonstrate properties of the presented controller design.

### 2.1. Available Signals

**Figure 1:**
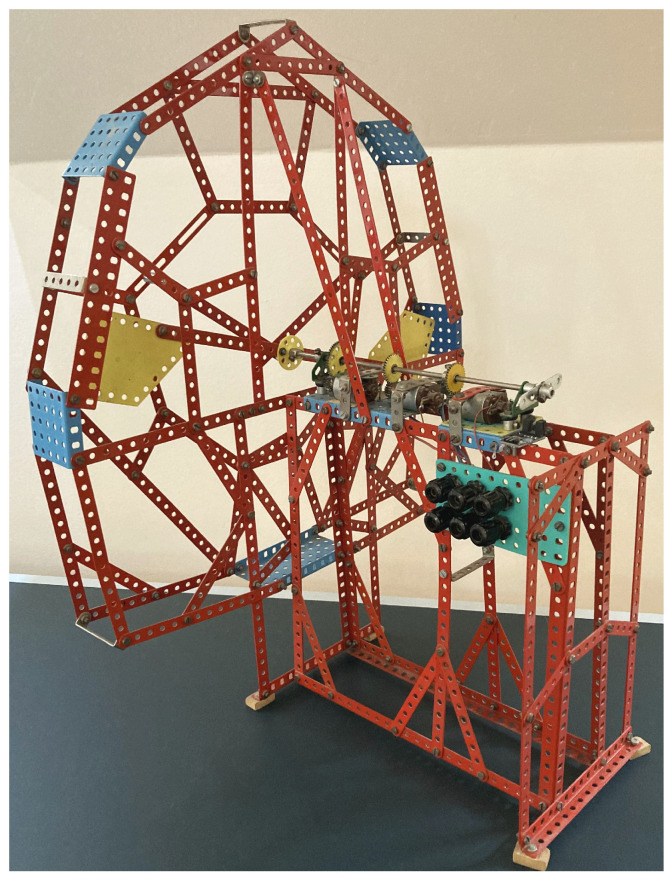
The considered electro-mechanical speed system.

All the controllers discussed in this paper will be illustrated by control of the speed servo system created by connecting three DC motors to one shaft with a high moment of inertia described in [4]. Two DCs in parallel serve as an actuator, the third as a sensor (tachodynamo) to measure the speed of rotation. For calibration, a simple incremental sensor has been used (Figure 1).

**Remark** **2** (Motivation to choose the electro-mechanical system). *Construction of the speed servo system created by connecting three DC motors to one shaft with a high moment of inertia was mainly motivated by low price, simple construction, simple connection to HP-85 computer converters, the dynamics with a dominant time constant close to electric vehicles and the possibility to create different functional configurations with the motors considered. The most recent addition to these historical aspects is the possibility to further develop the basic aspects of the design of series PI and PID controllers implemented by Arduino-Due’s controllers presented in [4]. The use of one of the motors as a speed sensor was already an obsolete option at the time of the system’s construction, and therefore, it was supplemented with an incremental sensor enabling at least system calibration. Of course, the spectrum of better quality sensors is much broader today and they need not be significantly more expensive (one of the basic historical motives for the birth of mechatronics was the aim to achieve the required quality of products at the lowest possible costs [34]). However, in the moment, when wishing to demonstrate the possibility of designing controllers with derivative action even for systems with a high level of measurement noise, the use of the motor in the tachodynamo function is even an advantage. The experience gained through experimentation can then be applied in the control of systems for which there exist no higher-quality sensors for the output measurement, or they are unavailable for the given solution.*

*It should also be noted that an application of the HO-PIDs derived for the IPDT model to control a stable nonlinear system, can also be considered as a robustness test of these controllers.*

Although the controller derivation assumes quasi-continuous-time control with pulse-amplitude modulation, in reality, the motor is controlled discretely using pulse-width-modulation (PWM) implemented with a sampling period of $T_{s}=10$ ms. The maximal available control signal corresponds to u=100, the minimal to u=0. When quantizing the pulse width, 256 possible levels are assumed, which are neglected in the design. All these differences due to the PWM control represent the first source of noise distorting the control process.

**Figure 2:**
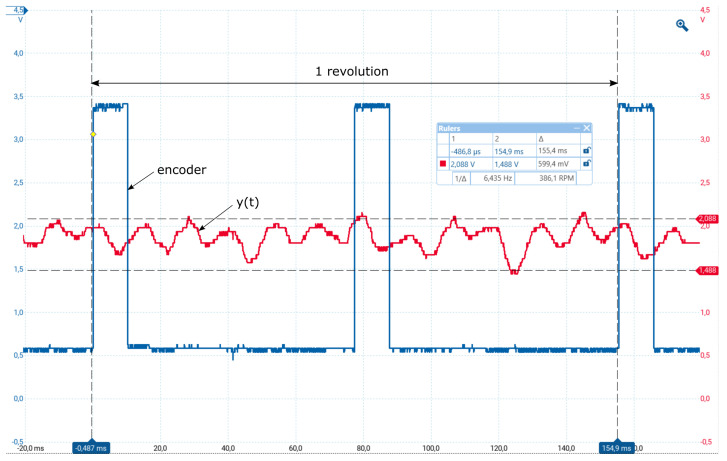
Two incremental sensor pulses (blue) specifying one revolution and the output velocity from tachodynamo with 12 maxima/minima per revolution (red), measured with $T_{sm}=0.1$ ms.

The output speed is measured by 12 bit sensors with the sampling period $T_{sm}=0.1$ ms, which again contributes to the measured signal as quantization noise. Measurement noise signals are also not negligible due to the variable resistance of the commutator contacts. In addition, the mechanical resonance of the shaft also contributes to the generation of noise and output fluctuations. However, the most important ripple of the measured signal (see Figure 2) is caused by the movement of the tachodynamo coils in an inhomogeneous magnetic field of the permanent magnet. The result is that the measurement noise amplitudes form a large part of the useful signal from the measurement range [0,1] (see Figure 2 in [4]). Due to this noise, the output signals fluctuate in the range of 0.5 V, which represents roughly 1/3 of the useful signal values.

### 2.2. The Experimental Setup

Due to the cheap motor used as the tachodynamo, i.e., a velocity sensor and other loop imperfections resulting in the high level of measurement noise, the measured output signal $y_{m}\left(t\right)$ of the considered velocity system must be filtered before the controller. Therefore, ref. [4] proposed to use binomial filters described with the transfer function

(1) $$\begin{array}{c} Q_{n}\left(s\right)=\frac{Y(s)}{Y_{m}\left(s\right)}=\frac{1}{{\left(T_{f}s+1\right)}^{n}}=\frac{a_{0}}{s^{n}+a_{n-1}s^{n-1}+\ldots +a_{1}s+a_{0}};\\ a_{k}=\frac{n!}{\left(n-k\right)!\;k!\;T_{f}^{n-k}};\;n=1,\;2,\;\ldots \;;\;k=0,1,2,\ldots ,n;\;\\ n!=n(n-1)(n-2)\;\ldots \;3*2*1 \end{array}$$

and integrated with the process in the step response-based process identification. Besides of the noise attenuation, it enabled to work in the controller design with ideal (acausal) controller transfer functions. For the choice n=4, motivated by the aim to improve noise attenuation (see, e.g., [30]), inclusion of the $Q_{4}\left(s\right)$ filter with $T_{f}=50$ ms into the process+filter approximation gave the ultra local IPDT model

(2) $$S\left(s\right)=\frac{Y(s)}{U(s)}=\frac{K_{s}e^{-T_{d}s}}{s};\;K_{s}=0.15;\;T_{d}=0.18$$

It should be remembered that this model aims to characterize the fastest component of transients as accurately as possible. It means that (2) represents an approximation with the maximum value of $K_{s}$ achieved from the multitude of different transients corresponding to numerous measured step responses. Among approximations with the maximum $K_{s}$, priority will be given to the solutions with the maximum value of $T_{d}$. While in [4], the need for an accurate approximation of the fastest component of the step response was more or less intuitive, the need for such a solution, when working with HO filters, was justified by the robust stability analysis in [6].

**Figure 3:**
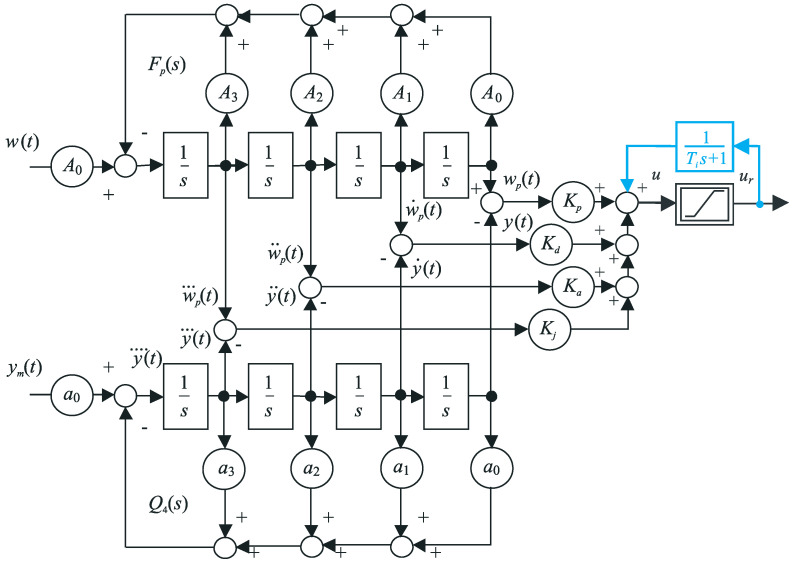
Series PIDAJ controller (34) with two degrees of freedom: the fourth-order filter $Q_{4}\left(s\right)$ (1) of the measured output $y_{m}\left(t\right)$, the automatic-reset with time constant $T_{i}$ (blue), with proportional, derivative, accelerative and jerk controller gains $K_{p}$, $K_{d}$, $K_{a}$ and $K_{j}$, with the reference setpoint w(t) and a fourth-order prefilter $F_{p}\left(s\right)$ (40).

Furthermore, it is of note that the fourth-order filter $Q_{4}\left(s\right)$ (see Figure 3) provides, in addition to the filtered output y(t), signals of its first, second and third derivatives.

**Remark** **3** (Motivation to use binomial strictly proper filters). *By default, in the design of PID control, as well as controllers with higher-order derivatives, separate filters of individual derivative components are mostly used. Thereby, they take the minimum order necessary to achieve a proper controller transfer function. With such an approach, with the growing number of derivatives, the number of unknown parameters will increase and, at the same time, the delays of the individual control components will change. Therefore, from the point of view of the analytical design of the optimal controller, it is simpler to consider a common binomial series filter.*

*From the point of view of the impact of noise, it is appropriate to work with strictly proper controller transfer functions. A simple experimental analysis in [30], and in several subsequent works, on the topic showed that*

(3) n≥m+2

*can be recommended, with equality considered as the default option.*

Whereas in [4], only y(t) and its first derivative $\dot{y}\left(t\right)$ were used by the series PI and PID controllers, next the full information provided by the filter, i.e., also the signals of the second and third derivative $\ddot{y}\left(t\right)$ and $\dddot{y}\left(t\right)$, will be used. If we were to use the fourth derivative $\ddddot{y}\left(t\right)$, then the measured values of the noise would not be filtered at all and, moreover, they would be further distorted by the feedback signal of the filter. The amplitudes of the noise contained in $\ddddot{y}\left(t\right)$ are gradually reduced by passing through four integrators through the averaging process.

Intuitively, one could expect that the use of derivatives will lead to a worsening of noise attenuation and result in an increase of the superimposed noise amplitudes. However, on the other side, one could also expect faster responses. Further questions also arise regarding the resulting robustness of the system. Let us not forget that already the arrival of hyper-reset (series PID) controllers using $\dot{y}\left(t\right)$ has proven to be a benefit for practice, even if it is accompanied with the increase in measurement noise. The cost function for controller optimization must therefore be built in such a way that it takes into account the compromise between the speed of transients and the excessive controller effort due to the noise and model imperfections. Therefore, it is interesting to test all these aspects experimentally and verify changes of the chosen *m* in individual performance measures.

### 2.3. Classical Versus State-Space Approach to Processes Approximated by IPDT Models

Within the framework of the state-space approach to the design of automatic controllers, systematic procedures of modern control theory [35,36] have been developed, when a stabilizing state controller is first considered for the given process. This is supposed to impose the required dynamics on the circuit.

In the next step, an extended state observer can be proposed, whose task is to directly reconstruct unmeasurable state variables and external disturbances related to the process model used in the controller design. The reconstructed disturbances have to be counteracted by a disturbance feedforward.

Within the framework of the classical approach to the design of controllers, on the other hand, more work was done with the input–output approximations of controlled processes using transfer functions, including typically the dead-time elements. Instead of the division of the overall task of controller design into the stabilization and reconstruction of non-measurable quantities, treated as disturbances, the design of proportional, integrative and derivative components of PID controller has been used, which were proclaimed to reflect the roles of past, present and future deviations of the output from the reference setpoint signal [37,38].

The unclear relation of the classical and modern approach to control led to the emergence of new postmodern approaches, e.g., internal model control (IMC) [39], active disturbance rejection control (ADRC) [40,41], model free control (MFC) [42], fractional-order (FO) PID control [9], etc. These, although they brought new, interesting moments, only further clouded the overall situation. At the same time, the constant growth of new control design methods brings serious problems for the field of education, where the overall space for teaching automatic control is generally reduced [43], and new serious aspects regarding the technological basis of control are added (increasingly wider use of various programmable devices and embedded systems, sensors, actuators, technical communications, etc.). From the point of view of reducing the ongoing fragmentation of the basics of automatic control, it is therefore extremely important to note that already the historical pneumatic automatic-reset and hyper-reset controllers, which can be considered as basic structures of industrial automation, can be treated as the structures with a stabilizing controller and disturbance observer (DOB). This DOB is reconstructing equivalent (or total) input disturbances in relation to integral process models from the evaluation of the controller output in steady states [7,8].

The first difference of automatic-reset, compared to structures from modern control theory, lies in the fact that the design of a controller with disturbance compensation changes the optimal setting of the stabilizing controller. It is a result of the positive feedback introduced by automatic-reset: the design of stabilization and disturbance compensation are not separable.

Another important aspect of comparing the classical and modern approaches to the controller design lies in the fact that the state-space approaches are usually not formulated for processes with dead-time, which in turn is a part of the most popular PID control design models as the integrator plus dead time (IPDT) or first-order time-delayed (FOTD) models [44]. The dead-time compensation has already been used in the proposal of extended state observer (ESO) in [45], but the work of [46] showed that this approach to its compensation is not the only possible one and it can be further improved.

One possibility to show the connections between the modern control theory and the PID control can be based on replacing the transport delay of the IPDT or FOTD model used in the PID controller design by expanding $exp(-T_{d}s)$ into a Taylor series [29].

Without the dead-time term, the integral first-order process model can be stabilized by considering a proportional (P) controller:

(4) $$R\left(s\right)=K_{p}$$

With the simplest replacement $exp\left(-T_{d}s\right)\approx 1/\left(1+T_{d}s\right)$, one gets the overall model of the process with a second-order transfer function. In the framework of the state-space controller design, its stabilization would correspond to a control vector with two entries. The corresponding state vector can also be transformed to a phase vector of the output formed by the output variable and its derivative, which in the classical design corresponds to a proportional-derivative (PD) controller with the proportional and derivative gains $K_{p}$ and $K_{d}$ and the derivative time constant $T_{D}$

(5) $$R\left(s\right)=K_{p}+K_{d}s=K_{p}\left(1+T_{D}s\right);\;K_{d}=K_{p}T_{D}$$

Similarly, it is possible to express the dead-time-term of IPDT and FOTD models by using two terms of its Taylor’s expansion. As, e.g., mentioned by [29], by considering the second-order Taylor approximation of dead-time, an improved future error estimation can be obtained and thus help in reducing the time delay by a predictive control signal calculation. This, in turn, leads to a control vector with three components, or to a classical controller with proportional, derivative and acceleration components (PDA controller)

(6) $$R\left(s\right)=K_{p}+K_{d}s+K_{a}s^{2}=K_{p}\left(1+T_{D}s+T_{A}^{2}s^{2}\right);\;K_{d}=K_{p}T_{D};\;K_{a}=K_{p}T_{A}^{2}$$

It has the proportional, derivative and accelerative gains $K_{p}$, $K_{d}$ and $K_{a}$, or the derivative and accelerative time constants $T_{D}$ and $T_{A}^{2}$.

Similar conclusions can be expected from the inclusion of the third- and higher-order derivatives of the delay. As the last option, we now mention just a controller used in motion control with proportional, derivative, acceleration and jerk feedback (PDAJ controller)

(7) $$\begin{array}{c} R\left(s\right)=K_{p}+K_{d}s+K_{a}s^{2}+K_{j}s^{3}=K_{p}\left(1+T_{D}s+T_{A}^{2}s^{2}+T_{J}^{3}s^{3}\right);\\ K_{d}=K_{p}T_{D};\;K_{a}=K_{p}T_{A}^{2};\;K_{j}=K_{p}T_{J}^{3} \end{array}$$

It has the gains of the proportional, derivative, accelerative and jerk actions $K_{p}$, $K_{d}$, $K_{a}$ and $K_{j}$. The controller can also be expressed by using time constants $T_{D}$, $T_{A}^{2}$ and $T_{J}^{3}$ of the derivative, accelerative and jerk actions.

The fact that all these solutions are only approximations should not surprise—also the IPDT and FOTD models are only approximations, and even far more sophisticated modeling and control design methods used in the framework of FO PID control [9] are ultimately only applied approximately. Thereby, in the simplest case, all above controllers can be based on the same process model (2), which ideally also includes the noise attenuation filter of the measured output. The central question of the experimental evaluation, which set of controller parameters, $\left\{K_{p}\right\}$, $\left\{K_{p},T_{D}\right\}$, $\left\{K_{p},T_{D},T_{A}^{2}\right\}$ or $\left\{K_{p},T_{D},T_{A}^{2},T_{J}^{3}\right\}$, ensures the best dynamics of the circuit, is not at all simple. We have not yet seen any attempts to deal with it. Each possible answer will certainly depend on the specification of optimal performance and measures used for the evaluation, but also on possible other aspects of the circuit, which are not directly included in the model (2). In our case, we simplified the question formulation by assuming a fixed structure of the filter $Q_{4}\left(s\right)$ (the order n=4, the time constant $T_{f}=50$ ms, the sampling period for filtration 0.1 ms), the same as in [4]. The basic difference is that in the controller in Figure 3, it is now possible to set up to four parameters $K_{p},K_{d},K_{a}$ and $K_{j}$. Depending on the stabilizing controller (4)–(7) used, the order and setting of the controller prefilter $F_{p}\left(s\right)$ also change.

### 2.4. Performance Measures

Development in the field of automatic control design is associated not only with discovery, but also with forgetting. In the case of PID control, this was manifested not only in the aforementioned forgetting of the original names automatic reset and hyper reset, but also in the abandonment of performance measures used in evaluating control performance. In the second half of the last century, as a result of wartime needs, there was a rapid development of constrained time-optimal systems. One of his main statements, labeled Feldbaum’s theorem, says that for optimal constrained control of transients in the output of *N*-th order systems, *N* control intervals with alternately changing limit values of the control signal are needed at its input [47]. If we limit ourselves to the transition from one steady state to another, this conclusion does not fundamentally change either Pontryagin’s principle of maxima (or minima), formulated later [48], nor Bellman’s principle of optimality [49]. Because the development of automatic control later went through periods in which issues of robustness dominated, especially in relation to linear systems, the conclusions of optimal constrained control were mostly forgotten during the rebirth of interest in PID control [50] (with few exceptions as [51,52]). With the constant acceleration of the dynamics of transients, the question of the influence of the control signal limitations on the ideal shape of the individual circuit variables cannot be avoided even here. However, before the practical application of Feldbaum’s theorem, it should yet be remembered that it is valid only for systems with a full relative degree R=N. When controlling systems with a lower relative order R<N, the number of necessary intervals to achieve the required output is reduced to *R*. The achieved output value can then be maintained using the so called “zeroing input” in the case of stable zero dynamics [53]. In practice, the impact on the control of stable systems is that the number of input intervals required for ideal output changes can be smaller than the total degree of the system and can depend on the designer’s choice. The selected dynamic class of control [54,55] then determines the selection of performance measures used to evaluate deviations from the input and output waveforms marked as optimal.

To obtain the transients as fast as possible, the absolute integral error

(8) $$IAE=\int_{0}^{\infty }\left|e(t)\right|dt\;\;;\;\;\;e=w-y$$

should be as low as possible. In evaluation of the setpoint step responses, the index “s” will be used. Similarly, for the input disturbance steps, the corresponding performance values will be denoted by the subscript “d”.

Similar to [4], evaluation of the shapes of the achieved step responses will be based on the monotonicity concept, with the monotonicity measure $TV_{0}$ and the one-pulse measure $TV_{1}$ defined for the increments of the setpoint and disturbance responses of the output y(t) as follows

(9) $$\begin{array}{c} TV_{0}\left(y_{s}\right)=\sum_{i=0}^{\infty }\left|y_{i+1}-y_{i}\right|-\left|y_{\infty }-y_{0}\right|\\ TV_{1}\left(y_{d}\right)=\sum_{i}\left|y_{i+1}-y_{i}\right|-\left|2y_{m}-y_{\infty }-y_{0}\right|;\;y_{m}\notin \left(y_{0},y_{\infty }\right) \end{array}$$

Thereby, $y_{m}$ in $TV_{1}\left(y_{d}\right)$ and similarly also $u_{m}$ in $TV_{1}\left(u\right)$

(10) $$TV_{1}\left(u\right)=\sum_{i}\left|u_{i+1}-u_{i}\right|-\left|2u_{m}-u_{\infty }-u_{0}\right|.$$

represent the extreme points used in enumerating deviations from two monotonic segments of one-pulse (1P) disturbance step responses at the process output and 1P setpoint and disturbance step responses at the process input (see, e.g., [4,56]).

**Remark** **4** (Performance evaluation of the time-delayed speed system). *In this work, we will consider ideal shapes at the system input (controller output) pulses consisting of two monotonic sections marked as 1P and we will quantify deviations from them using (10). From the point of view of the control of the selected speed system, this means that with a step increase in the required speed, only one pulse of energy supply at the input of the system will be expected, even if, as a result of the approximation of the delay, we are basically controlling a higher-order system, in which it would be possible to expect a more complex input course connected with kinetic energy reduction by braking just before reaching the required speed. Although the given method of control in systems with energy recuperation could also be implemented in an energy-saving manner, we will not deal with it further for relatively small delay values.*

## 3. Series PI, PID, PIDA and PIDAJ Controllers for IPDT Models

For the dead-time present in the loop, the design of stabilizing controllers can be carried out by the multiple real dominant pole (MRDP) method. The history of its use for the design of dynamic systems is roughly as long as the history of the automatic-reset controllers—it was already applied in the first textbook on automatic control [57], which also refers to its older use. MRDP tuning avoids slow and oscillatory components of the dynamics, which could dominantly limit achievable performance. For the design of constrained state-space controllers with extended state observers, which in today’s terminology could be denoted by the abbreviation ADRC, or as disturbance observer-based control, it was used in [58,59] (see also [46,60]). Vitecek and Viteckova [61,62] used the given method to design PI and PID controllers. The condition of a double real pole was also used by [31] in the modification of the SIMC method for design of PI and PID controllers for integral processes.

It should yet be noted that optimal setting of controllers augmented by automatic-reset differs from circuits containing only a stabilizing controller [7,8].

### 3.1. Calculation of the Optimal Series PI Parameters

For the P controller (4) augmented by automatic reset, in the loop with the IPDT model [4,5], it is possible to look for a triple real dominant pole $s_{o}$ of the quasi-polynomial $P\left(s\right)={\left(s-s_{o}\right)}^{3}P_{0}\left(s\right)$. Its parameters have to satisfy the conditions

(11) $$P\left(s_{o}\right)=0,\;\dot{P}\left(s_{o}\right)=0,\;\ddot{P}\left(s_{o}\right)=0$$

The corresponding closed loop pole $s_{o}$, the time constant $T_{o}=-1/s_{o}$ and the controller tuning specified by dimensionless parameters are

(12) $$\begin{array}{c} s_{o}=-\left(2-\sqrt{2}\right)/T_{d}\approx -0.586/T_{d};\;T_{o}=-\frac{1}{s_{o}}=T_{d}/\left(2-\sqrt{2}\right)\approx 1.71T_{d};\\ \kappa =K_{p}K_{s}T_{d}=2\left(\sqrt{2}-1\right)e^{\sqrt{2}-2}\approx 0.461;\;\tau_{i}=\frac{T_{i}}{T_{d}}=\left(2\sqrt{2}+3\right)\approx 5.828 \end{array}$$

To avoid overshooting of setpoint step responses, the controller has to be used with a first-order prefilter

(13) $$F_{p}\left(s\right)=\frac{1+bs}{1+T_{i}s}$$

introducing the second degree of freedom by canceling the numerator zero of the closed loop transfer function $F_{wy}\left(s\right)=R\left(s\right)S\left(s\right)/\left[1+R\left(s\right)S\left(s\right)\right]$. The setpoint step responses can be accelerated [63] by choosing the prefilter parameter *b* as

(14) $$b=T_{o}$$

thus also canceling one of the triple real dominant poles $s_{o}$ from $F_{wy}\left(s\right)$. However, in a loop with constrained control signal, the initial peak in the setpoint step of responses with b≠0 is usually removed by saturation. Therefore, it seems a more rational and robust option to use

(15) $$b=0;\;F_{p}\left(s\right)=\frac{A_{0}}{s+A_{0}};\;A_{0}=1/T_{i}$$

### 3.2. Calculation of the Optimal Series PID Parameters

For the PD controller (5) augmented by automatic-reset [4,5], in the loop with the IPDT model, it is possible to look for a quadruple real dominant pole $s_{o}$ of the quasi-polynomial $P\left(s\right)={\left(s-s_{o}\right)}^{4}P_{0}\left(s\right)$, by satisfying the conditions

(16) $$P\left(s_{o}\right)=0,\;\dot{P}\left(s_{o}\right)=0,\;\ddot{P}\left(s_{o}\right)=0,\;\dddot{P}\left(s_{o}\right)=0$$

The corresponding closed loop pole $s_{o}$, the time constant $T_{o}$ and the controller tuning specified by dimensionless parameters are

(17) $$\begin{array}{c} s_{o}=-\left(3-\sqrt{3}\right)/T_{d}\approx -1.268/T_{d};\;T_{o}=-\frac{1}{s_{o}}=\frac{T_{d}}{3-\sqrt{3}}\approx 0.789T_{d};\\ \kappa =K_{p}K_{s}T_{d}\approx 0.0598;\;\tau_{i}=\frac{T_{i}}{T_{d}}\approx 0.2846;\;\\ \tau_{D}=\frac{T_{D}}{T_{d}}\approx 3.448;\;\delta =K_{d}K_{s}=\kappa \tau_{D}=0.2062 \end{array}$$

Compared to the PI controller (12), by using the derivative action, the dominant pole $s_{o}$ shifted further to the left in the complex plane and the corresponding closed loop time constant $T_{o}$ decreased significantly. Of the two possible numerator factorizations of the controller transfer function (see [4]), we chose the dimensionless gain κ and the feedback constant $\tau_{i}$ as smaller numbers, with increased $\tau_{D}$. This makes it possible to speed up the dynamics of constrained responses during the transition from a limited control signal to a steady-state value and prevent the occurrence of overshoots at the input and output of the process [4,5].

To avoid overshooting of setpoint step responses, the controller has to be used with a second-order prefilter

(18) $$F_{p}\left(s\right)=\frac{1+b_{1}s+b_{2}s^{2}}{\left(1+T_{i}s\right)\left(1+T_{D}s\right)}$$

canceling at least the numerator zero of the closed loop transfer function $F_{wy}\left(s\right)=R\left(s\right)S\left(s\right)/\left[1+R\left(s\right)S\left(s\right)\right]$. The setpoint step responses can yet be accelerated by choosing the prefilter numerator $N_{p}\left(s\right)$ as

(19) $$N_{p}\left(s\right)={\left(1+T_{o}s\right)}^{p};\;p\in \left[0,2\right]$$

that is canceling *p* of the quadruple real dominant poles $s_{o}$. Again, especially in constrained control, the most robust option can be recommended, which corresponds to the numerator tuning with p=0, yielding

(20) $$b_{1}=b_{2}=0;\;F_{p}\left(s\right)=\frac{A_{0}}{s^{2}+A_{1}s+A_{0}};\;A_{0}=\frac{1}{T_{i}T_{D}};\;A_{1}=\frac{T_{i}+T_{D}}{T_{i}T_{D}}$$

### 3.3. MRDP-Based Calculation of the Optimal Series PIDA Parameters

A more complicated situation arises from the factorization of the obtained controller transfer function point of view in the case of the PIDA controller.

By introducing a disturbance observer in the form of a low-pass filter $1/(1+T_{i}s)$ connected to the (possibly saturated) overall controller output and compensating the reconstructed disturbance at the output of the stabilizing PDA controller, in the proportional zone of control, the series PIDA controller transfer function can be expressed as

(21) $$R\left(s\right)=\frac{\left(K_{p}+K_{d}s+K_{a}s^{2}\right)\left(1+T_{i}s\right)}{T_{i}s}=K_{p}\frac{\left(1+T_{D}s+T_{A}^{2}s^{2}\right)\left(1+T_{i}s\right)}{T_{i}s}$$

For a nominal IPDT process (2), it yields the closed-loop transfer function

(22) $$F_{c}\left(s\right)=\frac{R(s)S(s)}{1+R(s)S(s)}=\frac{\left(K_{s}K_{a}s^{2}+K_{s}K_{d}s+K_{s}K_{p}\right)\left(1+T_{i}s\right)}{s^{2}T_{i}e^{T_{d}s}+\left(K_{s}K_{a}s^{2}+K_{s}K_{d}s+K_{s}K_{p}\right)\left(1+T_{i}s\right)}$$

with the characteristic quasi-polynomial

(23) $$A\left(s\right)=s^{2}T_{i}e^{T_{d}s}+\left(K_{s}K_{a}s^{2}+K_{s}K_{d}s+K_{s}K_{p}\right)\left(1+T_{i}s\right)$$

Four unknown PIDA controller parameters, together with the position of the unknown dominant pole $s_{o}$ represent five unknown parameters that can be calculated by considering quintuple dominant pole fulfilling

(24) $${\left[d^{i}A\left(s\right)/ds^{i}\right]}_{s=s_{o}}=0\;;\;\;i=0,1,2,3,4$$

From an equation formulated by using the fourth A(s) derivative as $d^{4}A\left(s\right)/ds^{4}=0$, it is now possible to calculate the dominant pole/time constant

(25) $$s_{o}=-\left(4-\sqrt{4}\right)/T_{d}=-2/T_{d};\;\;\;T_{o}=-1/s_{o}=T_{d}/2$$

The dimensionless controller parameters can be calculated from (24) (e.g., by using a computer algebra support) as

(26) $$\begin{array}{c} \kappa =K_{p}K_{s}T_{d}\approx 0.9323;\;\tau_{i}=\frac{T_{i}}{T_{d}}\approx 2.5832;\;\tau_{D}=\frac{T_{D}}{T_{d}}\approx 0.4168;\;\\ \delta =K_{d}K_{s}=\kappa \tau_{D}\approx 0.3885;\tau_{A}^{2}=\frac{T_{A}^{2}}{T_{d}^{2}}\approx 0.0484;\;\alpha =\frac{K_{a}K_{s}}{T_{d}}=\kappa \tau_{A}^{2}\approx 0.04511 \end{array}$$

To avoid overshooting of setpoint step responses, the controller has to be used with a third-order prefilter

(27) $$F_{p}\left(s\right)=\frac{1+b_{1}s+b_{2}s^{2}+b_{3}s^{3}}{\left(1+T_{i}s\right)\left(1+T_{D}s+T_{A}^{2}s^{2}\right)}$$

canceling the numerator zeros of the closed loop transfer function (22). The setpoint step responses can yet be accelerated by choosing the prefilter numerator $N_{p}\left(s\right)$ (19) with p∈[0,3] to cancel *p* of the quintuple real dominant closed-loop poles $s_{o}$. Again, the most robust option corresponds to the numerator tuning with p=0, when

(28) $$\begin{array}{c} b_{1}=b_{2}=b_{3}=0;\;\\ F_{p}\left(s\right)=\frac{A_{0}}{s^{3}+A_{2}s^{2}+A_{1}s+A_{0}};\;\\ A_{0}=\frac{1}{T_{i}T_{A}^{2}};\;A_{1}=\frac{T_{i}+T_{D}}{T_{i}T_{A}^{2}};\;A_{2}=\frac{T_{i}T_{D}+T_{A}^{2}}{T_{i}T_{A}^{2}} \end{array}$$

The PIDA controller parameters κ and $\tau_{i}$ (27) are now obviously closer to the series PI controller (12) than to the series PID ones (17). This is due to the fact that the R(s) numerator

(29) $$N_{PIDA}=1+T_{D}s+T_{A}^{2}s^{2}=1+0.4168T_{d}s+0.0484T_{d}^{2}s^{2}$$

does not have real poles that could alternate with the calculated value $T_{i}$ during R(s) factorization, but the complex conjugate pole-pair

(30) $$s_{1,2}=\left(-4.305785124\pm 1.456492874j\right)/T_{d},$$

The impossibility of choosing a shorter time constant $T_{i}$ from the controller parameters complying with (24) will cause the more delayed transition from a control limit to the steady state. Thus, in the case of transient responses with a constrained control signal, the transients will be slowed down, with the subsequent occurrence of process input and output overshoots. Work [28] therefore proposed to replace $N_{PIDA}$ by neglecting the imaginary parts of its poles, i.e., approximating it with a double real pole/time constant as

(31) $${\bar{N}}_{PIDA}=T_{A}^{2}s^{2}+T_{D}s+1\approx 0.0538s^{2}T_{d}^{2}+0.4640T_{d}s+1={\left(0.2320T_{d}s+1\right)}^{2}$$

Subsequently, the controller transfer function (21) with one set of parameters (26) and $N_{PIDA}$ replaced by ${\bar{N}}_{PIDA}$ has to be factorized for the new time constant $T_{i}=0.2320T_{d}$ (see the equivalent conversion for series PID control in [4]) as follows:

(32) $$\begin{array}{c} T_{i}=0.2320T_{d};\;K_{p}=0.932273\frac{0.232}{2.583K_{s}T_{d}}=\frac{0.0837}{K_{s}T_{d}};\\ T_{D}=2.583T_{d}+0.232T_{d}=2.815T_{d};\;K_{d}=K_{p}T_{D}=0.2356/K_{s};\\ T_{A}^{2}=\left(2.583T_{d}\right)\;0.232T_{d}=0.599T_{d}^{2};\;K_{a}=T_{A}^{2}K_{p}=0.0502T_{d}/K_{s} \end{array}$$

This yields the new set of dimensionless parameters

(33) $$\begin{array}{c} \kappa =K_{p}K_{s}T_{d}\approx 0.0837;\;\tau_{i}=\frac{T_{i}}{T_{d}}\approx 0.2320;\;\tau_{D}=\frac{T_{D}}{T_{d}}\approx 2.815;\;\\ \delta =K_{d}K_{s}=\kappa \tau_{D}\approx 0.2356;\tau_{A}^{2}=\frac{T_{A}^{2}}{T_{d}^{2}}\approx 0.599;\;\alpha =\frac{K_{a}K_{s}}{T_{d}}=\kappa \tau_{A}^{2}\approx 0.0502 \end{array}$$

In comparable items, these recalculated parameters are already much closer to the optimal PID (17) than the parameters of the original PIDA controller (26). Thereby, comparing to PID, the recalculated κ value increased slightly, the $\tau_{i}$ and $\tau_{D}$ values decreased. This approximate design with a reduced $T_{i}$ value shows smoother responses with a minimum number of monotonic segments at the process output. Similarly as the series PIDA controller (26), also the design based on the parallel PIDA controller tuned with the MRDP method and modified with anti-windup based on the conditioning technique [27,33] leads to constrained transients with input and output overshoots, which is not acceptable in numerous mechatronic applications.

### 3.4. MRDP-Based Calculation of the Series PIDAJ Parameters

By introducing a disturbance observer in the form of a low-pass filter $1/(1+T_{i}s)$ connected to the (possibly saturated) overall controller output and compensating the reconstructed disturbance at the output of the stabilizing PDAJ controller (see Figure 3), in the proportional zone of control, the series PIDAJ controller transfer function can be expressed as

(34) $$R\left(s\right)=\frac{\left(K_{p}+K_{d}s+K_{a}s^{2}+K_{j}s^{3}\right)\left(1+T_{i}s\right)}{T_{i}s}=K_{p}\frac{\left(1+T_{D}s+T_{A}^{2}s^{2}+T_{J}^{3}s^{3}\right)\left(1+T_{i}s\right)}{T_{i}s}$$

For a nominal IPDT process (2), it yields the closed loop transfer function

(35) $$F_{c}\left(s\right)=\frac{R(s)S(s)}{1+R(s)S(s)}=\frac{\left(K_{s}K_{j}s^{3}+K_{s}K_{a}s^{2}+K_{s}K_{d}s+K_{s}K_{p}\right)\left(1+T_{i}s\right)}{s^{2}T_{i}e^{T_{d}s}+\left(K_{s}K_{j}s^{3}+K_{s}K_{a}s^{2}+K_{s}K_{d}s+K_{s}K_{p}\right)\left(1+T_{i}s\right)}$$

with the characteristic quasi-polynomial

(36) $$A\left(s\right)=s^{2}T_{i}e^{T_{d}s}+\left(K_{s}K_{j}s^{3}+K_{s}K_{a}s^{2}+K_{s}K_{d}s+K_{s}K_{p}\right)\left(1+T_{i}s\right)$$

Five unknown PIDAJ controller parameters, together with the position of the unknown dominant pole $s_{o}$, represents six unknown parameters that can be calculated by considering the six-fold dominant pole. From an equation formulated by using the fifth A(s) derivative as $d^{5}A\left(s\right)/ds^{5}=0$ it is now possible to calculate the dominant pole/time constant as

(37) $$s_{o}=-\left(5-\sqrt{5}\right)/T_{d}\approx -2.7639/T_{d};\;\;\;T_{o}=-1/s_{o}\approx 0.3618T_{d}$$

The dimensionless controller parameter can now be calculated as

(38) $$\begin{array}{c} \kappa =K_{p}K_{s}T_{d}\approx 0.0915;\;\tau_{i}=\frac{T_{i}}{T_{d}}\approx 0.1747;\;\\ \tau_{D}=\frac{T_{D}}{T_{d}}\approx 2.4434;\;\delta =K_{d}K_{s}=\kappa \tau_{D}\approx 0.2236;\\ \tau_{A}^{2}=\frac{T_{A}^{2}}{T_{d}^{2}}\approx 0.7166;\;\alpha =K_{a}K_{s}/T_{d}=\kappa \tau_{A}^{2}\approx 0.0656\\ \tau_{J}^{3}=\frac{T_{J}^{3}}{T_{d}^{3}}\approx 0.0709;\;\gamma =K_{j}K_{s}/T_{d}^{2}=\kappa \tau_{J}^{3}\approx 0.0065 \end{array}$$

To avoid overshooting of setpoint step responses, the controller has to be used with a prefilter

(39) $$F_{p}\left(s\right)=\frac{1+b_{1}s+b_{2}s^{2}+b_{3}s^{3}+b_{4}s^{4}}{\left(1+T_{i}s\right)\left(1+T_{D}s+T_{A}^{2}s^{2}+T_{J}^{3}s^{3}\right)}$$

canceling the numerator zero of the closed loop transfer function (35). The setpoint step responses can be accelerated by choosing the prefilter numerator $N_{p}\left(s\right)$ (19) with p∈[0,4] to cancel *p* of the quintuple real dominant closed-loop poles $s_{o}$. Again, the most robust option corresponds to the numerator tuning with p=0, yielding

(40) $$\begin{array}{c} b_{1}=b_{2}=b_{3}=b_{4}=0;\;\\ F_{p}\left(s\right)=\frac{A_{0}}{s^{4}+A_{3}s^{3}+A_{2}s^{2}+A_{1}s+A_{0}};\\ A_{0}=\frac{1}{T_{i}T_{J}^{3}};\;A_{1}=\frac{T_{i}+T_{D}}{T_{i}T_{J}^{3}};\;A_{2}=\frac{T_{i}T_{D}+T_{A}^{2}}{T_{i}T_{J}^{3}};\;A_{3}=\frac{T_{i}T_{A}^{2}+T_{J}^{3}}{T_{i}T_{J}^{3}} \end{array}$$

## 4. Experimental Comparison

In the experimental comparison of the series PI, PID, PIDA and PIDAJ controllers that all can be based on the $Q_{4}\left(s\right)$ filter specified in [4], we will start from the IPDT model of system (2), which was found with the aim of the best possible approximation of the fastest mode of the measured setpoint step response. Because this model provides the largest possible value of $K_{s}$, according to [28], we expect it to provide the best possible robust stability areas even when using higher-order filters (in our case, n=4). This filter provides all the necessary signals to verify the above-mentioned controllers.

**Figure 4:**
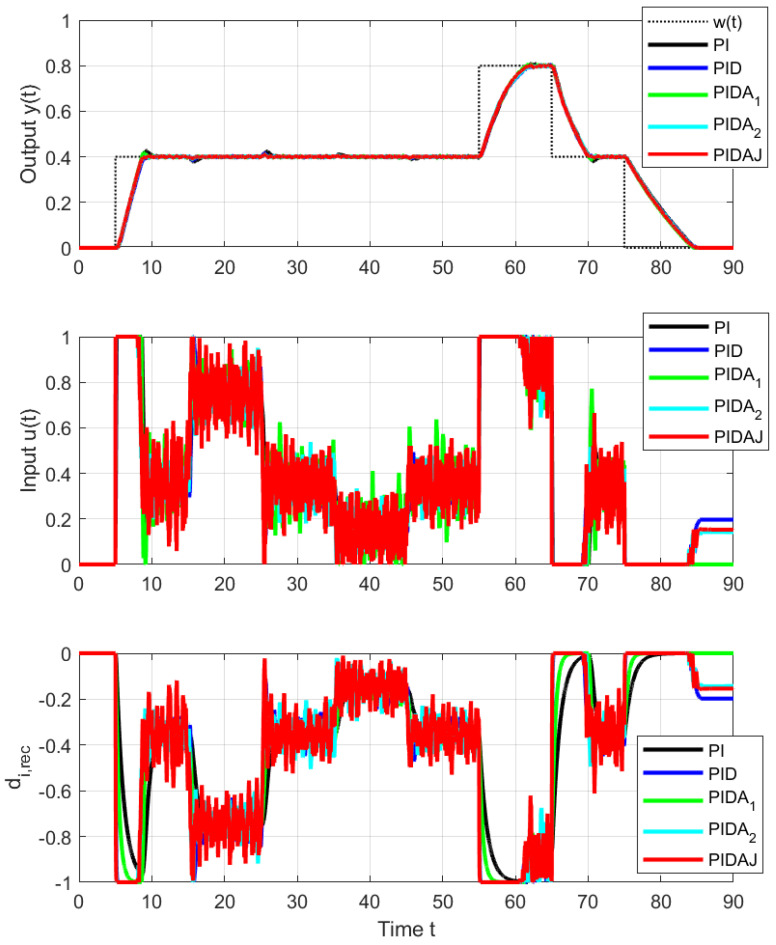
Measured signals from the experiment corresponding to: setpoint step to w=0.4 at t=5 s, load step Δu=0.5 at t∈[15,25) s, load step Δu=0 at t∈[25,35) s, load step Δu=−0.25 at t∈[35,45) s, load step Δu=0 at t∈[45,55) s, setpoint step to w=0.8 at t=55 s, to w=0.4 at t=65 s and to w=0 at t=75 s.

When organizing the experiment, steps of the reference setpoint signal and steps of the input disturbance (load) have been considered. Because (especially with respect to the control signal limitations) asymmetries tend to appear in real loops, setpoint and disturbance steps are realized both upwards and downwards (Figure 4).

### 4.1. Setpoint Step Responses

**Figure 5:**
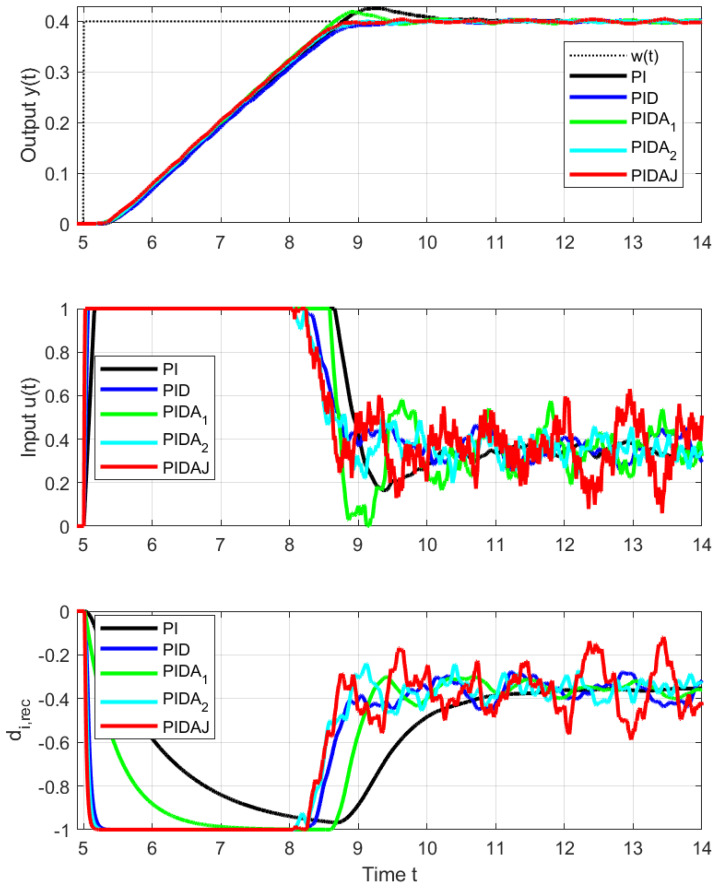
Details of setpoint step responses from w=0 to w=0.4 at t=5 s.

**Figure 6:**
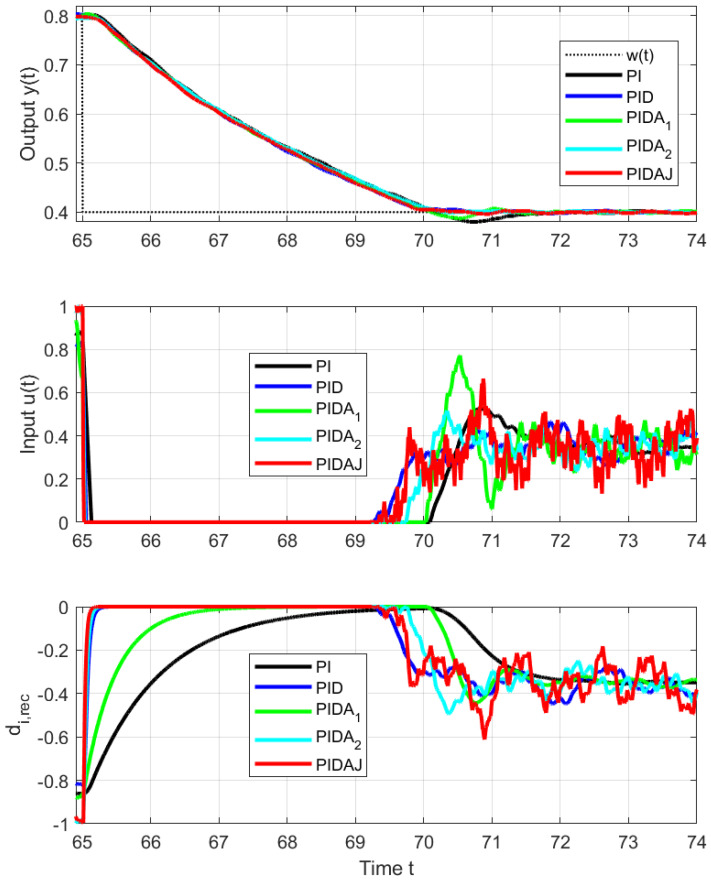
Details of setpoint step responses from w=0.8 to w=0.4 at t=65 s.

As in the IPDT system control, setpoint step responses corresponding to PI and PIDA${}_{1}$ controller appear with overshoot (see Figure 5 and Figure 6), which is the largest for the PI controller. The corresponding performance measures computed for steps towards w=0.4 up and down are in Table 1. The excessive effort of the controller increases with the derivative degree *m*.

In view of the fact that we are actually controlling a stable system, the overshoot of PI and PIDA${}_{1}$ control (signaled mainly by increased PO and $TV_{0}\left(y\right)$ values) is smaller than would correspond to an IPDT system, but still big enough to deal with. The size of the setpoint steps in Figure 5 and Figure 6 upwards and downwards is the same. However, due to the different limit values of the control signal and contribution to asymmetry caused by the internal feedback influencing the equivalent load disturbance, they will not exceed the setpoint in the same way: upward step responses are faster (smaller $IAE_{s}$ values), and all shape deviations are larger.

The use of controllers with higher-order derivatives, in general, leads to a reduction of the initial overshoot PO through the setpoint value and to an acceleration of transient responses, including disturbance reconstruction. However, it leads simultaneously to increased oscillation of the output and the input around the steady states (increasing $TV_{1}\left(u\right)$ values). For the PIDAJ controller, the amplitude of permanent oscillations is already greater than the initial overshoot caused by limiting the control signal during the transient response. Therefore, when it comes to the smoothest possible alignment of transient responses and steady states, we obtain a result similar to conclusion [30]—the order of the used filter should be chosen as n≥m+2.

### 4.2. Disturbance Step Responses

**Figure 7:**
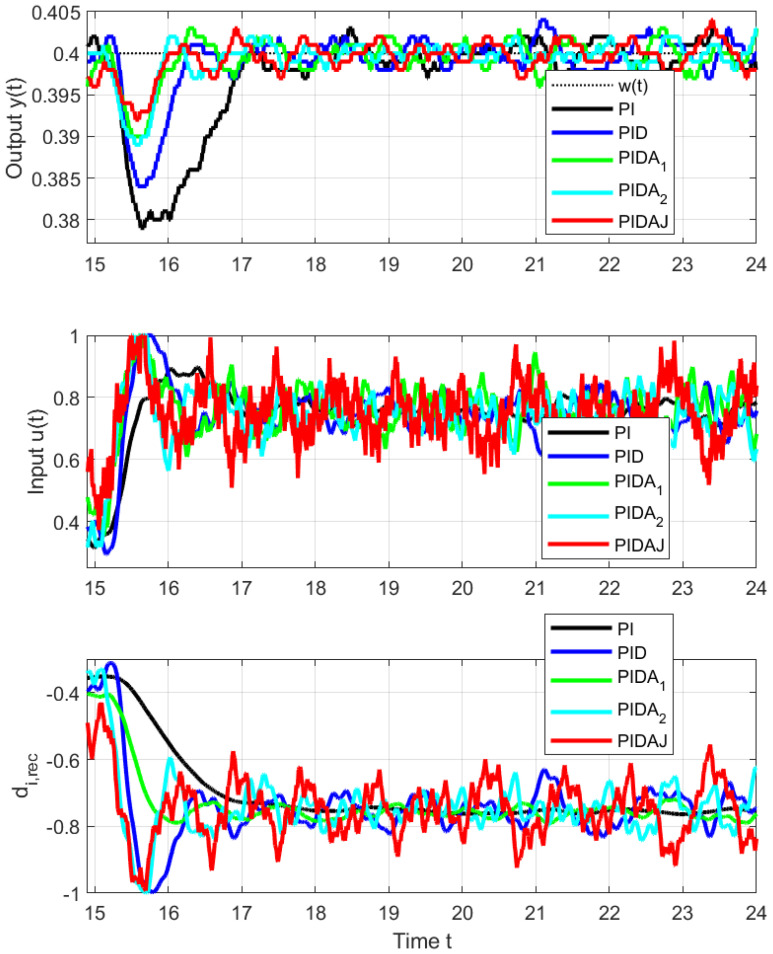
Details of load disturbance step responses from Δu=0 to Δu=0.5 at t=15 s.

**Figure 8:**
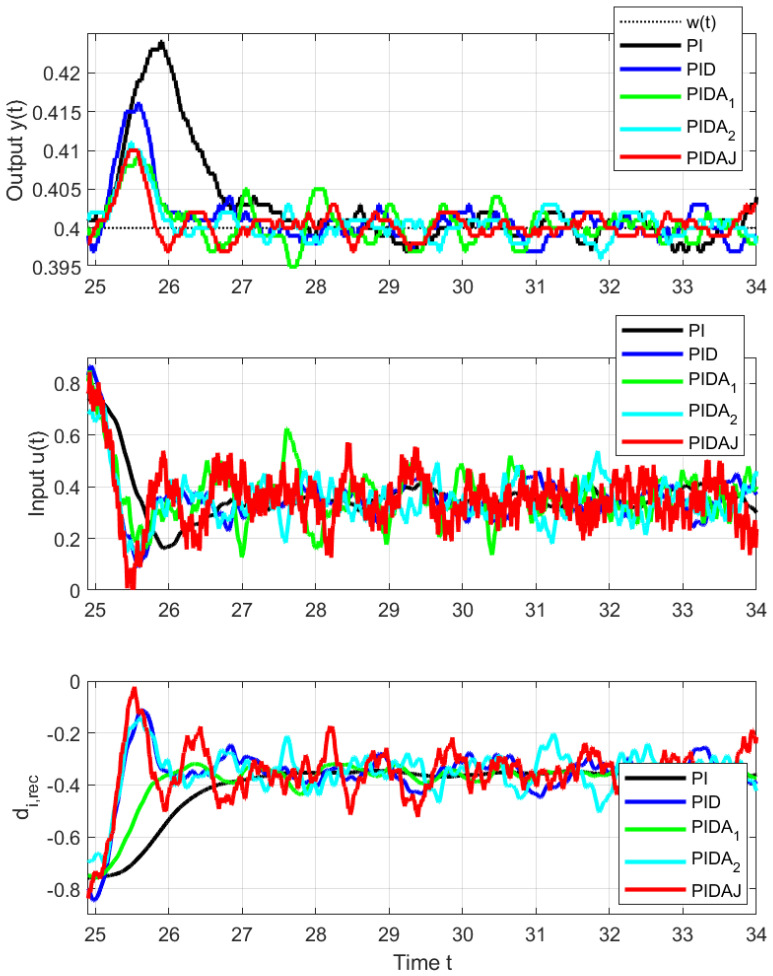
Details of load disturbance step responses from Δu=0.5 to Δu=0 at t=25 s.

Since the disturbance steps for t∈[35,55) did not bring new information, just the disturbance steps carried out on the interval t∈[15,35) will be evaluated. Figure 7 and Figure 8 and the data in Table 2 correspond to upwards and downwards steps implemented by an additional load signal steps superimposed to the controller output. In this case, the asymmetry of the jumps up and down is not as great as with setpoint steps, because the corresponding range of output changes is now significantly smaller. However, the effect of increasing the order of the derivative degree *m* used in controller on speeding up transients and reducing the maximum overshoot due to a disturbance step is uniform and clearly positive.

The signal of the reconstructed disturbance is also established faster. From the disturbance reconstruction point of view, however, the performance of controllers with higher-order derivatives is more debatable, because they lead to more oscillating signals, which may still require additional filtering to yield some information about the disturbance acting on the process.

## 5. Discussion

In Section 2, we presented an IPDT model of a DC motor with a fourth-order filter at the controller input. In it, we addressed the question of what types of controllers can be used based on the signals provided by the given control structure. In [4], the choice was limited to the use of PI or PID controllers. After the introduction of PIDA and PIDAJ controllers, the most optimal (limit) results were obtained with higher-order controllers. The conclusions from the experimental verification can be interpreted in different ways. For example, for the required speed of transient responses (IAE) or minimum shape deviations at the input and output of the process, the specified performance can be achieved by designing a suitable filter (changing n and Tf). Once the filter with order n is determined, the most optimal (limit) performance can correspond to a controller structure with derivatives of even higher-order than the PIDAJ controller. Analogous to FO PID control [9], the use of controllers with higher-order derivatives leads to the creation of new degrees of freedom in the design.

### 5.1. Filter Design

In [4], the selection of the $Q_{4}$ filter was motivated by the requirement to have sufficiently filtered controller output signals even for PID controller. This was achieved by choosing a sufficiently high relative degree of the controller with filter ($n_{r}=3$). In this work, all output derivatives used by the controller were generated with the same $Q_{4}$ filter, which subsequently simplified the analytical design of the optimal loop parameters. This approach is innovative compared to the alternative approaches [11,12,13,14,15,16,17,18,19,20,21,22,23,24,64], where the output derivatives (or accelerations) are generally computed using separate filters for each controller derivative term or for the entire controller, but without additional information about the effect of the filter on the process identification.

**Figure 9:**
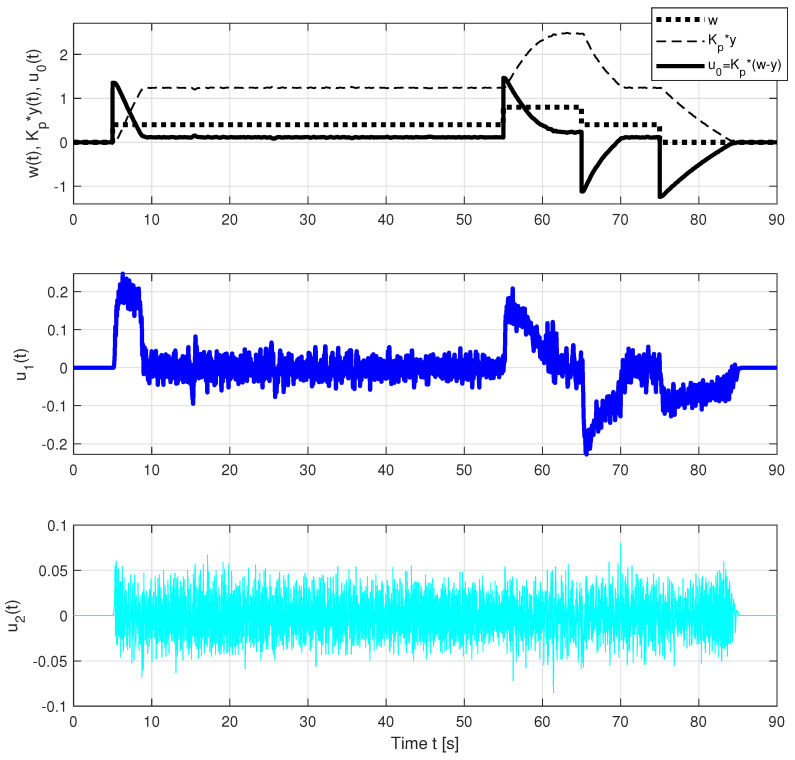
Individual control components of the PIDA controller corresponding to different control error derivatives and the transients according to Figure 4.

**Figure 10:**
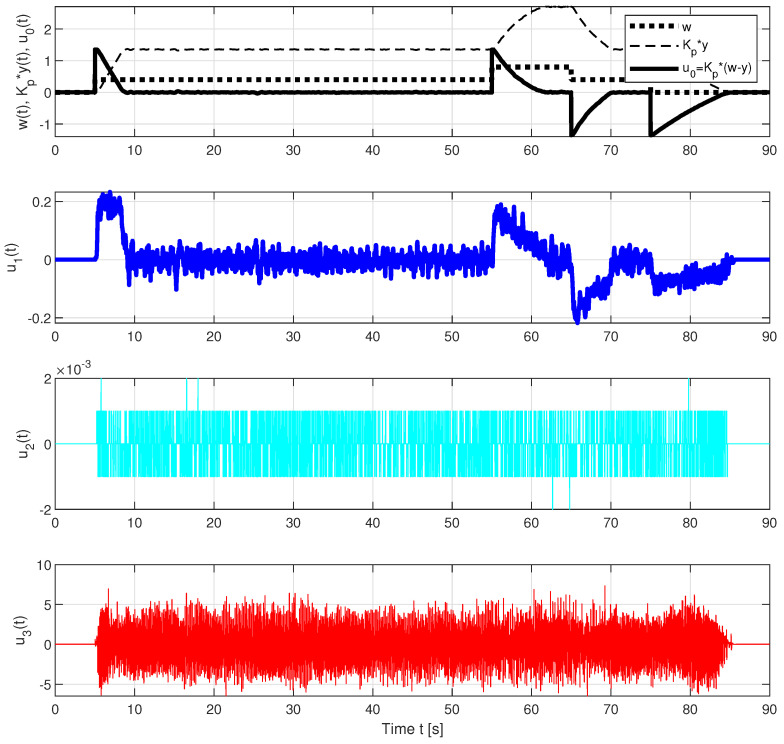
Individual control components of the PIDAJ controller corresponding to different control error derivatives and the transients according to Figure 4.

The problems associated with the use of strictly proper controllers can be illustrated by comparing the responses of the individual components of the PIDA and PIDAJ controllers in Figure 9 and Figure 10. The controllers are strictly proper with relative degrees 2 and 1. However, the jerk feedback of the PIDAJ controller (which does not meet recommendation (3)) already acquires significant values that can be called excessive controller effort. Their amplitudes are significantly higher than the contribution of the proportional component $u_{0}\left(t\right)=K_{p}\left(w\left(t\right)-y\left(t\right)\right)$.

Thus, the PIDA and PIDAJ controllers are calculated for the process approximated by (2) according to (33) and (38):

(41) $$\begin{array}{c} \mathrm{PIDA}:\;T_{i}=0.0418;\;K_{p}=3.1000;\;K_{d}=1.5707;\;K_{a}=0.0602;\;\\ \mathrm{PIDAJ}:\;T_{i}=0.0314;\;K_{p}=3.3889;\;K_{d}=1.4907;\;K_{a}=0.0787;\;K_{j}=0.0014. \end{array}$$

exhibit relatively low derivatives of the control error. However, as shown in Table 1 and Table 2, even coefficients with such small values can lead to significant improvement in transient response.

Because automatic feedback reduces the control error, the value of the proportional component $u_{0}\left(t\right)$ gradually decreases after abrupt changes in w(t), even as the output value itself, and hence the value of $K_{p}y\left(t\right)$, increases. At low output values, the contribution of the system’s internal feedback is negligible. At the upper limit of the control signal, the output value changes almost linearly, as in the control of an integral system. At higher output levels, the internal feedback of the system (the actual system is not the integrating, but a stable process) is already dominant and the output changes gradually slower while the limit of the control signal is still active.

The derivative component $u_{1}\left(t\right)=K_{D}\left(\dot{w}-\dot{y}\right)$ is also practically constant for small values of the process output and the limited process input signal. At high output values, the derivative component decreases exponentially. The internal feedback of the system slows down the transient response.

The acceleration component of the control $u_{2}\left(t\right)=K_{A}\left(\ddot{w}-\ddot{y}\right)$, in the PIDA controller (satisfies (3)) is relatively small compared to $u_{0}$ and $u_{1}$. In the PIDAJ controller, the changes are relatively small and are around zero. Such an influence may seem insignificant, but in the control of unstable systems, the acceleration component allows a wider range of delays [32].

The jerk component of the PIDAJ controller with the filter applied (not satisfying (3)) can be considered a waste of energy, even though this component further extends the allowable delay values of unstable processes [32]. However, the sharp increase in the amplitude of this signal suggests that if the derivative component order were further increased and the relative controller degree is 0, yet higher excessive noise would occur at the controller output. A similar conclusion regarding the degree of derivatives used and filter degree applies more generally (see Remark 3).

It should be noted that acting disturbances at stabilizing controller outputs are practically imperceptible.

### 5.2. Series Versus Parallel HO-PID Controllers

As an extension of the traditional series PI and PID controllers, the newly introduced design of PIDA and PIDAJ controllers tuned by the MRDP method expands the range of available closed-loop dynamics in saturating control. However, similar to the previous studies [4,5,28], it requires a specific factorization of the controller transfer function with the selection of $T_{i}$ as the smallest numerator time constant. If the numerator contains complex zeros, the factorization can be done by approximately replacing the given pair with a double real zero. Although the parallel (see [27]) and series structures lead to the same transient responses in linear control, the responses differ significantly when the control signal is limited. The parallel PI, PID, PIDA and PIDAJ control with traditional anti-windup solutions based on the conditioning technique [33] resulted in overshoot of the output for the given controller structures with reference filters [27], while the newly proposed series solutions with modified controller transfer functions had no or negligible overshoot. It should also be noted that a direct implementation of the conditioning technique is not possible, since it requires strictly proper controllers. Therefore, the order of the filters should be reduced to obtain a strictly proper controller, and the remaining filter can be placed after the control limitations. Thus, the proposed controller design using a modification of the MRDP method enabled a simple realization of controllers that yield near-optimal control loop dynamics under the given controller structures.

However, since parallel controllers may give better results in some situations, the design and comparison of series and parallel controllers, or the consideration of different factorizations of series controllers, must still be treated as an open problem.

### 5.3. Trade-Off in Automatic-Reset Tuning

The task of calculating the optimal value of $T_{i}$ is a typical trade-off that occurs in engineering applications. With respect to the reconstruction of disturbances from the steady-state values of the controller output, $T_{i}$ must be as long as possible. On the other hand, for a fast transition from the saturation limit to a steady-state value of the control signal, $T_{i}$ must be short. To analyze the effect of $T_{i}$ in combination with the nonlinear saturation block (which is a special case of sectoral nonlinearity), the control loop must be transformed into a canonical equivalent circuit with saturation and a linear part [28,52,65,66,67]. However, in such a case, different controller parameterizations correspond to different transfer functions of the linear part, which could explain the different behavior.

### 5.4. Robustness Versus Excessive Control Effort

The decrease in IAE values when the derivative degree (*m*) in the controllers is increased is associated with an increase in $TV_{1}\left(u\right)$ values. However, it should be recalled that robust controllers are often implemented as sliding mode control (SMC) [68,69,70,71,72], where the control signal is constantly oscillating, which can lead to a sharp increase in $TV_{1}\left(u\right)$. It should be noted that even these sliding controllers can provide smooth, well-damped control signal waveforms in the limit case. From this point of view, the evaluation results are also not a surprise, but only a revelation of existing trends by showing some relationships between HO PIDs and SMCs.

## 6. Conclusions

Combining classical and modern control approaches, the design of control structures with automatic-reset for reconstruction and compensation of constant input disturbances is presented. Thereby, the stabilizing controllers are used with higher-order derivatives. It is shown that by adding to them a positive feedback in the form of a low-pass filter from the entire controller output, a simple disturbance observer (DOB) is achieved that reliably reconstructs constant disturbances at the process input by evaluating the steady-state values of the possibly limited controller output.

The automatic tracking-based DOB and stabilizing controllers with derivatives have already been derived up to the tenth order. However, in this work, we restrict ourselves to the third-order derivatives provided by the noise attenuation filter $Q_{4}\left(s\right)$ (1), which is considered as part of the process in [4]. With respect to the robustness of the higher-order filters, the process identification should aim at obtaining the IPDT model with the largest possible values of the gain $K_{s}$ and $K_{s}T_{d}$ (see [6]).

To obtain the optimal estimate of the loop dynamics, which is also suitable for the constrained control, the time constants of the controller numerator are calculated using the multiple real dominant method [4,28] and subsequently factorized. The time constant for automatic reset is then chosen as the shortest possible time constant of the controller numerator.

It was shown in [4] that although it is possible to approximate a stable speed servo system more accurately using FOTD models, the use of simpler IPDT models is at least equivalent in terms of control performance. Since the computational complexity increases with increasing derivative degree, especially when designing PIDA and PIDAJ controllers, we restricted ourselves to IPDT models. The results obtained were in line with expectations, showing that closed-loop velocities increase and overshoot decreases as the derivative degree of the controller increases.

Although this brand new family of generalized hyper-reset-based controllers is related to the HO PID controllers proposed in [27], they are equivalent only in the linear domain, which becomes very narrow, especially when higher-order derivatives are used. The remaining question is whether it is still appropriate to refer to both families of controllers by a single name [73].

Future work in this area will focus on the following:

- A more detailed investigation of possible design improvements for even higher derivative degrees;
- The question of when an increase in derivation order is appropriate in practice;
- Further investigation of the relationship between series and parallel controllers, solutions with different disturbance observers, comparisons with fractional order PIDs;
- HO controllers based on double integrator and dead time models.

## Figures and Tables

**Table 1:** Performance measures IAE, $TV_{0}\left(y\right)$ and $TV_{1}\left(u\right)$ corresponding to the setpoint steps from Figure 5 and Figure 6 calculated for $t_{1}\in \left[5,15\right)$, $y\left(t_{1}\right)=y_{1},u\left(t_{1}\right)=u_{1},w\left(t_{1}\right)=w_{1}=0.4$, $t_{2}\in \left[65,75\right)$, $y\left(t_{2}\right)=y_{2},u\left(t_{2}\right)=u_{2},w\left(t_{2}\right)=w_{2}=0.4$ with maximal (red) and minimal values (blue), $PO_{1},PO_{2}$—percentage overshoot.

	${\mathrm{IAE}}_{1}$	${\mathrm{TV}}_{0}\left(y_{1}\right)$	${\mathrm{TV}}_{1}\left(u_{1}\right)$	${\mathrm{PO}}_{1}$	${\mathrm{IAE}}_{2}$	${\mathrm{TV}}_{0}\left(y_{2}\right)$	${\mathrm{TV}}_{1}\left(u_{2}\right)$	${\mathrm{PO}}_{2}$
PI	**0.853**	0.100	**1.208**	**6.50**	**0.948**	0.074	**0.934**	**5.00**
PID	0.852	0.066	3.170	0.75	0.903	0.048	2.774	0.75
$PIDA_{1}$	0.820	**0.122**	7.932	4.75	0.915	**0.082**	6.338	3.25
$PIDA_{2}$	0.813	**0.060**	8.244	**0.50**	0.921	**0.046**	6.012	**0.50**
PIDAJ	**0.809**	0.098	**27.198**	1.25	**0.901**	0.060	**22.288**	1.00

**Table 2:** Performance measures IAE, $TV_{1}\left(y\right)$ and $TV_{1}\left(u\right)$ corresponding to the load disturbance steps from Figure 7 and Figure 8 calculated for $t_{1}\in \left[15,25\right)$, $y\left(t_{1}\right)=y_{1},u\left(t_{1}\right)=u_{1},w\left(t_{1}\right)=w_{1}=0.4$, $t_{2}\in \left[25,35\right)$, $y\left(t_{2}\right)=y_{2},u\left(t_{2}\right)=u_{2},w\left(t_{2}\right)=w_{2}=0.4$ with maximal (red) and minimal values (blue), PO—percentage overshoot.

	${\mathrm{IAE}}_{1}$	${\mathrm{TV}}_{1}\left(y_{1}\right)$	${\mathrm{TV}}_{1}\left(u_{1}\right)$	${\mathrm{PO}}_{1}$	${\mathrm{IAE}}_{2}$	${\mathrm{TV}}_{1}\left(y_{2}\right)$	${\mathrm{TV}}_{1}\left(u_{2}\right)$	${\mathrm{PO}}_{2}$
PI	**0.032**	**0.082**	**1.426**	**5.25**	**0.034**	**0.080**	**1.452**	**6.00**
PID	0.020	0.106	4.962	4.00	0.019	0.104	4.756	4.00
$PIDA_{1}$	0.015	**0.130**	11.584	2.50	0.019	**0.150**	11.630	2.25
$PIDA_{2}$	**0.014**	0.104	11.512	2.75	0.015	0.112	11.276	2.75
PIDAJ	0.015	0.112	**42.386**	**2.00**	**0.014**	0.100	**43.820**	**2.50**

## Data Availability

Not applicable.

## Abbreviations

The following abbreviations are used in this manuscript:

1P	One-Pulse, response with 2 monotonic segments (1 extreme point)
AW	Anti-Windup
ADRC	Active Disturbance Rejection Control
FO	Fractional Order
HO	Higher Order
IAE	Integral of Absolute Error
IFAC	Integral Federation of Automatic Control
IMC	Internal Model Control
IPDT	Integrator Plus Dead-Time
MRDP	Multiple Real Dominant Pole
P	Proportional
PD	Proportional-Derivativee
PDA	Proportional-Derivative-Accelerative
PDAJ	Proportional-Derivative-Accelerative-Jerk
PI	Proportional-Integral
PID	Proportional-Integral-Derivative
PIDA	Proportional-Integral-Derivative-Accelerative
PIDAJ	Proportional-Integral-Derivative-Accelerative-Jerk
PPM	Performance Portrait Method
PWM	Pulse-Width-Modulated
SMC	Sliding Mode Control
$TV_{0}$	Deviation from Monotonicity
$TV_{1}$	Deviation from 1P Shape
